# Systematic drug screening reveals specific vulnerabilities and co-resistance patterns in endocrine-resistant breast cancer

**DOI:** 10.1186/s12885-016-2452-5

**Published:** 2016-07-04

**Authors:** Sara Kangaspeska, Susanne Hultsch, Alok Jaiswal, Henrik Edgren, John-Patrick Mpindi, Samuli Eldfors, Oscar Brück, Tero Aittokallio, Olli Kallioniemi

**Affiliations:** Institute for Molecular Medicine Finland (FIMM), Biomedicum 2U, Tukholmankatu 8, 00290 Helsinki, Finland; Present address: Helsinki Innovation Services, Tukholmankatu 8 A, 00290 Helsinki, Finland; Present address: MediSapiens Ltd, Erottajankatu 19B, 00130 Helsinki, Finland; Present address: Science for Life Laboratory, Department Oncology-Pathology, Karolinska Institutet, Tomtebodavägen 23, 171 65 Solna, Sweden

**Keywords:** Tamoxifen resistance, Breast cancer, High-throughput drug testing, Exome-sequencing, Drug resistance

## Abstract

**Background:**

The estrogen receptor (ER) inhibitor tamoxifen reduces breast cancer mortality by 31 % and has served as the standard treatment for ER-positive breast cancers for decades. However, 50 % of advanced ER-positive cancers display *de novo* resistance to tamoxifen, and acquired resistance evolves in 40 % of patients who initially respond. Mechanisms underlying resistance development remain poorly understood and new therapeutic opportunities are urgently needed. Here, we report the generation and characterization of seven tamoxifen-resistant breast cancer cell lines from four parental strains.

**Methods:**

Using high throughput drug sensitivity and resistance testing (DSRT) with 279 approved and investigational oncology drugs, exome-sequencing and network analysis, we for the first time, systematically determine the drug response profiles specific to tamoxifen resistance.

**Results:**

We discovered emerging vulnerabilities towards specific drugs, such as ERK1/2-, proteasome- and BCL-family inhibitors as the cells became tamoxifen-resistant. Co-resistance to other drugs such as the survivin inhibitor YM155 and the chemotherapeutic agent paclitaxel also occurred.

**Conclusion:**

This study indicates that multiple molecular mechanisms dictate endocrine resistance, resulting in unexpected vulnerabilities to initially ineffective drugs, as well as in emerging co-resistances. Thus, combatting drug-resistant tumors will require patient-tailored strategies in order to identify new drug vulnerabilities, and to understand the associated co-resistance patterns.

**Electronic supplementary material:**

The online version of this article (doi:10.1186/s12885-016-2452-5) contains supplementary material, which is available to authorized users.

## Background

Breast cancer is the most common cancer in women worldwide. Two-thirds of breast tumors express ER that drives proliferation of mammary epithelial cells and thereby contributes to the etiology and progression of the disease. Consequently, antagonists that directly block ER function or drugs that lower the amounts of the natural ligand of ER, estradiol, have been utilized in breast cancer treatment for decades [1]. For over 40 years, tamoxifen, a selective ER antagonist, has been the backbone in treating ER-positive breast cancers. Despite of being effective in decreasing mortality, *de novo* or acquired resistance frequently occurs [2]. Some of the mechanisms leading to resistance have been revealed, including mutations in the gene encoding ER [3–5], altered expression patterns of ER or its cofactors [6, 7], and crosstalk between ER and growth factor receptor cascades such as the EGFR/ERK1/2-pathway [8]. Consequently, inhibition of ERK1/2 has been reported to restore antiestrogen sensitivity. For example, a study with the MEK inhibitor PD098059, a compound that reduces the phosphorylation and activation of ERK1/2, was shown to inhibit the growth of tamoxifen-resistant cell lines and to restore their sensitivity to therapy [9, 10]. However, ERK1/2 inhibition has proven efficacy primarily against cells with resistance-provoked overexpression or activation of HER2 [9]. On the other hand, recent findings suggest that proteasome inhibition might offer a new avenue for overcoming endocrine resistance [11, 12]. Bortezomib, a proteasome inhibitor, has been investigated as a combination therapy in conjunction with endocrine treatment in a phase II study [13].

Whilst shRNA- or cDNA-based functional screens [14, 15] and candidate gene [16–19], or drug [9, 20–23] approaches have been used to study the development and reversal of endocrine resistance, the exact molecular mechanisms remain unknown, and large-scale studies on cells treated long-term with tamoxifen are lacking. Moreover, efforts to find new treatment regimes for overcoming drug resistance have been largely based on a few selected drug candidates, and have only proven to be effective in a fraction of the cases [1]. Development of primary drug resistance can make the cancer cells susceptible for novel vulnerabilities, hence leading to additional therapeutic opportunities. However, secondary resistances towards other drugs may also arise. Resistance to chemotherapeutics has been linked with estrogen receptor positive breast cancer [24], but systematic studies on tamoxifen resistance associated co-resistances have not been conducted. Therefore, systematic, large-scale studies to characterize the drug sensitivity profiles of tamoxifen-resistant breast cancer are warranted to reveal new drug vulnerabilities as well as co-resistance patterns in drug-resistant cells.

Here, we report the development and characterization of a panel of seven long-term tamoxifen-treated breast cancer cell lines from four parental strains. Using these resistant cell line models and their isogenic parental counterparts, we, for the first time, performed systematic high throughput drug sensitivity and resistance testing with 279 approved and investigational oncology drugs to reveal potential new drug vulnerabilities and to identify co-resistance patterns acquired with tamoxifen resistance. We further conducted exome-sequencing on each of the isogenic parental-resistant cell line pair to identify point mutations and copy number variations that may contribute to drug resistance. Through integrated network analyses, we uncovered cell- and clone-specific molecular and functional patterns of endocrine resistance, highlighting the underlying molecular diversity, and pointing to several distinct therapeutic opportunities to circumvent it. However, no systematic drug screens with hundreds of oncology compounds on acquired tamoxifen resistance have been conducted.

## Methods

### Cell culture

Human breast cancer cell lines MCF-7 (HTB-22, ATCC), T-47D (HTB-133, ATCC), ZR-75-1 (CRL-1500, ATCC) and BT-474 (HTB-20, ATCC) were obtained from the American Type Culture Collection. The cells were grown in DMEM with L-Glutamine (MCF-7 and BT-474, PAN Biotech, Aidenbach, Germany) or RPMI-1640 with L-Glutamine (ZR-75-1 and T-47D, PAN Biotech) supplemented with 10 % FCS (Gibco, Life Technologies, Carlsbad, CA) and 1 % penicillin/streptomycin (Gibco). Culture media for T-47D, MCF-7 and BT-474 additionally contained 0,1 % bovine insulin (Sigma. St. Louis, MO). The tamoxifen-resistant cell lines (MCF-7 Tam1, T-47D Tam1 & Tam2, ZR-75-1 Tam1 & Tam2, BT-474 Tam1 & Tam2) were derived from the parental cell lines by continuous exposure to 4-OH-tamoxifen (Sigma, 1 μM in ethanol) for 8–12 months. Culture media was replaced every 2–3 days. All cells were incubated at 37 °C with 5 % CO_2_ and passaged when ca 80 % confluent. The approximate doubling times of the cells were as follows: parental MCF-7, T-47D, ZR-75-1 and BT-474: 1–3 days. Resistant MCF-7 Tam1, T-47D Tam1 and Tam2: 1–2 weeks, ZR-75-1 Tam1 and Tam2: > 1 week, BT-474 Tam1 and Tam2: 2 weeks. The cells were free of mycoplasma and verified for their authenticity (Technology Centre, Institute for Molecular Medicine Finland, Helsinki, Finland).

### Characterization of tamoxifen-resistant cell lines

For viability measurements cells were seeded in 384-well culture plates with increasing tamoxifen concentrations (0–1,8 μM). After 120 h cell viability was evaluated by CellTiter-Glo Cell Viability Assay (Promega, Fitchburg, WI) with the PHERAstar plate reader (Agilent Technologies Santa Clara, CA). To measure the active DNA synthesis cell proliferation assays with Click-iT® EdU Alexa Fluor® 488 Flow Cytometry Assay Kit were performed according to manufacturer’s protocol (Life Technologies) with following minor modifications: Cells were plated on 10 cm plates and cultured with and without 1 μM tamoxifen until approximately 50 % confluent. Parental cells and their tamoxifen-resistant derivatives were then pulsed with 10 μM of EdU Alexa Fluor® 488 for 4 h (T-47D and MCF-7) or for 28 h (BT-474 and ZR-75-1). Cells were permeabilized with saponin-based permeabilization and wash reagent (MCF-7, T-47D, BT-474) or with 0,1 % TritonX-100-PBS (ZR-75-1) for 10 min. Additionally, DNA content staining was performed using FxCycle™ Far Red and the cell suspension treated with RibonucleaseA. Flow cytometry was carried out and results analyzed using Accuri C6 flow cytometer and associated software (BD Biosciences, Franklin Lakes, NJ). To measure estrogen-responsivity of the parental cells and their tamoxifen-resistant derivatives, the cells were grown on 6-well plates in hormone-deprived culture medium for 72 h (phenol red-free DMEM or RPMI, PAN Biotech), supplemented with 2,5 % dextran–charcoal-treated (Sigma-Aldrich) FCS and other additives (see above). 17β-estradiol (Sigma, 10^−8^ M in ethanol) was then added back to the cells for 4, 8 or 24 h and RNA isolated with Total RNA Purification kit (Norgen, Thorold, ON). 4 μg of total RNA were reverse transcribed with the High-Capacity cDNA Reverse transcription kit (Applied Biosystems, Thermo Scientific, Waltham, MA) as instructed. Quantitative-PCR was then performed on the LightCycler 480 system (Roche, Penzberg, Germany) using the DyNAmo colour flash SYBRGreen PCR kit (Thermo Scientific) with equal amounts of cDNA. The optimal internal reference gene was determined out of a pool of 16 different housekeeping genes for each parental-resistant cell line pair (*18S* for MCF-7 s, *PPIA* for T-47Ds and *B2M* for ZR-75-1 s and BT-474 s). Primer sequences can be found in Additional file 1. All experiments were done in triplicates. For Western Blotting cells were grown on 10 cm dishes, and lyzed in Laemmli buffer. Immunoblotting was done as previously described [25]. The used antibodies were as follows: ERα (Abcam, Cambridge, UK, ab16660), β-actin (Sigma-Aldrich, A1978), EGFR (Cell Signaling Technologies, CST4267), phospho-EGFR (CST3777), ERK1/2 (CST9107), phospho-ERK1/2 (CST4370). To test the effects the ERK inhibitor VX-11E and the MEK inhibitor selumintinib the cells were cultured either in their default culture media with no additional drug, or with increasing concentrations of VX-11E (50nM, 100nM and 250nM), or with 100nM VX-11E in combination with 1 μM selumetinib. The cells were then harvested and Western blotting with the above-mentioned antibodies performed.

### Genomic profiling by exome-sequencing

Genomic DNA was isolated from the parental and tamoxifen-resistant cells using the DNeasy Blood & Tissue kit (Qiagen, Venlo, Netherlands and Hilden, Germany). Exome-capture was done on 3 μg DNA with the NimbleGen SeqCap EZ Human Exome v2.0 kit (Roche NimbleGen) and paired-end sequencing performed on Illumina HiSeq platform. Point mutations were detected as previously described [26], using the parental cell lines as controls. Briefly, point mutations specific to resistant samples were called with VarScan2 somatic [27], with the following parameters: strand-filter 1, min-coverage-normal 8, min-coverage-tumor 6, somatic-*p*-value 1, normal-purity 1, min-var-freq 0,05. Parental cell line was used as the normal control. Mutation calling was done within the exome capture target regions of the NimbleGen SeqCap EZ v2 capture kit and the flanking 500 bps. Mutations were annotated with SnpEff [28] using the Ensembl v66 annotation database. To filter out misclassified germline variants, common population variants included in dbSNP version 135 were removed. Remaining non-synonymous mutations were visually validated using the Integrated Genomics Viewer (Broad Institute). Mutations with *p* < 0,05 and resistant variant frequency >30 % were deemed high confidence. Known false positive point mutations [29] were excluded. Exome-sequencing data was also analyzed using the sequence alignment and variant calling pipeline VCP. As input in the CNV analysis we used alignments in BAM format, as well as identified variants in all samples [30] (and unpublished). All exome sequencing capture kit target regions less than 76 bp apart were merged with each other. An RPKM (reads per kilobase of target region length per million mapped reads) copy number value was calculated separately for every target region, followed by filtering out regions with sequencing coverage lower than 25x. Finally, relative log2 copy number ratios for sample (drug-resistant variant) divided by reference (parental cell line) were calculated and segmented using Circular Binary Segmentation [31]. Plots of copy number, segmentation and variant allele frequencies in capture target regions were visualized using R. Gene level copy number data for all human genes in Ensembl database v67 was calculated by assigning a gene the value of the CNV data segment that it overlapped. When a gene overlapped more than one segment, the gene was assigned a copy number value based on a modification of the extreme method option in GISTIC2 [32–34] as follows: the gene was given the lowest segment log2 value in case any overlapped segment had log2 ratio < = −0,6 and the highest segment value if any overlapped segment had log2 ratio > = 0,5. If all segments the gene overlapped had log2 ratio > −0,6 and < 0,5; the gene was assigned the median log2 ratio of all overlapped segments. Thresholds for copy number changes were determined based on samples (not published here) with known copy number differences, such as male vs female comparisons on chromosome X, as well as trisomies observed in karyotyping of cells during routine diagnostics. Based on these, the limits were set at −0,4 (heterozygous deletion), −1,2 (homozygous deletion), +0,5 (gain) and +1,3 (amplification). Raw exome-sequencing data have been deposited in the NCBI Sequence Read Archive [SRP: SRP050366].

### Drug sensitivity and resistance testing (DSRT)

DSRT with the FIMM FO2Baq library containing 279 approved and investigational oncology drugs was done as previously described [26] with minor modifications. Briefly, drugs were dissolved and plated in five different concentrations covering a 10 000-fold concentration range into the wells of 384-well plates. Optimized amounts of cells were then seeded into the wells in their normal growth media, i.e. parental cells in normal media and tamoxifen-resistant cells in media supplemented with 1 μM 4-OH-tamoxifen. Thus, this set-up allows for measurement of permanent drug response changes corresponding to long-term tamoxifen treatment used in the clinical setting, and allows for any combinatorial drug responses to be observed. A further Cells were incubated at 37 °C for 72 h and viability measured by CellTiter-Glo Cell Viability Assay with the PHERAstar plate reader. Data were normalized to negative (DMSO only) as well as positive (100 μmol/l benzethonium chloride) controls. The logistic dose–response curves were estimated using the Marquardt-Levenberg algorithm and implemented in the in-house bioinformatic pipeline Breeze. The dose–response curves were then employed to quantitatively profile drug responses, i.e. the Drug Sensitivity Score (DSS), which is based on the estimated logistic model parameters, and the DSS difference, which quantifies the differential drug response between tamoxifen-resistant and parental cells, as previously described [26, 35]. We found that |dDSS| = 5 cutoff lies in the tail end of the distribution with 9.7 % of values above the cutoff. With a two-tailed distribution (signed DSS), this would correspond to ca. 5 % “hit rate”, which we deemed as appropriate for such a drug discovery approach. Clustering of the DSS response differences across resistant/parental cell line pairs was performed using unsupervised hierarchical complete-linkage clustering, using Spearman and Euclidean distance measures of the drug and sample profiles, respectively, and visualized as a heat map [35]. In order to identify drugs that significantly change their efficacy as the cells gain resistance to tamoxifen, we performed rank product analysis [36] at false discovery rate of 5 % (q < 0,05) by comparing drug response profiles in the parental cells against response profiles in the tamoxifen-resistant cells. The average DSS activity of a drug in all parental cell lines was plotted against the average DSS activity in the resistant clones. The drugs selected from the rank product analysis were considered as significant hits and displayed as colored dots. Luminal A or B subtype-specific drugs were identified (Additional file 2) based on the known subtypes of the parental cells [37].

### Construction of drug sensitivity and co-resistance networks

To visualize drug sensitivity and resistance networks in each resistant/parental cell line pair, drugs with DSS difference >5 (sensitivity) or < −5 (co-resistance) were selected. For each drug, specific target molecules were defined using the KIBA (Kinase Inhibitor BioActivity) -score [38] as follows: First, drug target bioactivity (Ki, Kd and IC_50_) values were extracted from the ChEMBL database (https://www.ebi.ac.uk/chembl), and integrated KIBA-score was calculated for each drug-target pair. A low KIBA-score indicates a high binding affinity of the drug with the target. Amongst the set of targets for a drug, if the target with highest binding affinity had KIBA-score of <0,1, the specific target threshold was considered to be 50-fold the lowest KIBA-value; otherwise KIBA-score <3 units was considered as the specific target threshold. Second, to capture the connection between genomic changes and drug-target genes of the most effective drugs in each resistant cell model compared to its parental cell line, network analysis and visualization was done on KIBA-scored specific targets genes of the sensitizing or desensitizing drugs using Ingenuity Pathway Analysis application (Ingenuity® Systems, Qiagen). The IPA system is based on the Ingenuity Pathways Knowledge Base, which is a repository of curated biological interactions, and functional annotations between proteins, genes, complexes extracted from scientific articles. The network consists of thousands of nodes and the edges represent experimentally observed cause-effect relationships that are related to direct physical interactions or regulatory interactions events like expression, transcription, activation and molecular modifications. Network edges are also associated with a direction of the causal effect, i.e. either activating or inhibiting [39]. Sensitizing and desensitizing drugs were considered separately. We required each resistant/parental cell line pair to have at least three effector drugs with a DSS difference >5 to enable network building based on their target genes, discarding the sensitivity networks for MCF-7 Tam1 and ZR-75-1 Tam1 as each of them had one effector drug only. The target molecule networks representing the most effective sensitizing or desensitizing drugs were merged, and then adjusted to keep the edges that were human-specific and high confidence. The resulting networks were then extended to upstream neighboring molecules to reveal connections with genes containing copy number variations or somatic mutations within the target networks. In the IPA framework, upstream molecules are defined as the genes that have been shown to affect the gene expression in some direct/indirect way [39]. The IPA network generation algorithm considers all the immediate upstream molecules separated by one degree from the nodes in the current network, where priority is given to those genes having maximum number of overlaps with the existing network. Furthermore, we merged the IPA canonical pathway for Estrogen Receptor Signaling (www.qiagen.com) with the networks utilizing the IPA overlay tool.

## Results

### Development and characterization of long-term tamoxifen-treated cell lines

**Fig. 1:**
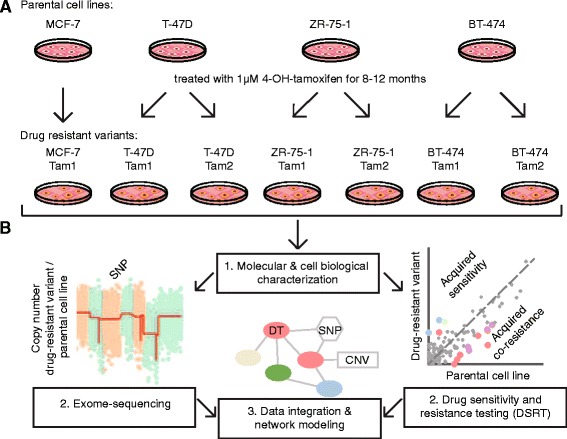
Development and analyses of the parental breast cancer cell lines and their tamoxifen-resistant variants. **a** Schematic representation of the four parental cell lines (MCF-7, T-47D, ZR-75-1 and BT-474) and their seven tamoxifen-resistant variants (arrows) that were developed through continuous tamoxifen-treatment. **b** Followed by molecular and cell biological characterization (1), drug sensitivity and resistance testing and exome-sequencing to detect point mutations (single nucleotide polymorphisms, SNP) and copy number variations (CNV) (2), the data was then integrated and the pharmacogenomic relationships visualized through network modeling (3). DT = Drug target. To reveal drug responses and genetic changes associated with tamoxifen resistance, all analyses were performed by comparing each tamoxifen-resistant cell line to its parental control cell line

To study the development of tamoxifen resistance across distinct molecular backgrounds, we established seven long-term tamoxifen-treated cell lines originating from four parental cell types; MCF-7, T-47D, ZR-75-1 and BT-474. We exposed these ER-positive, initially tamoxifen-responsive cells to continuous 1 μM 4-OH-tamoxifen treatment (hereafter tamoxifen) for 8 to 12 months (Fig. 1). A steady concentration of tamoxifen with clear inhibitory effect on ER-mediated transcription was chosen to mimic the exposure of patients to the drug in the clinic. In the course of the treatment, all cell cultures underwent cell death leaving a few founder cells that then recovered and repopulated the culture plates. Once stable, the cultures were molecularly characterized and subjected to pharmacogenomic profiling through drug sensitivity and resistance testing (DSRT) and exome-sequencing (Fig. 1). To confirm the resistant phenotypes, we assessed the viability of the cells upon increasing tamoxifen concentrations. All resistant cells showed increased tolerance towards tamoxifen compared to the parental cells, verifying the resistance development (Additional file 3A). For further characterization, we studied the cell cycle properties, functionality and levels of ER, and estrogen-responsivity of the long-term tamoxifen-treated cells. Resistant cell clones exhibited altered cell cycle properties, with a higher fraction of cells in the G0/G1 phase (Additional file 3B). All resistant cells had diminished ER target gene transcription, whereas the level of ER itself either increased or decreased depending on the clone (Additional file 4). Estradiol (E2) deprivation and subsequent addition of estradiol back to the cells revealed aberrant levels of ER target gene transcription, indicating that upon acquiring resistance to the ER antagonist tamoxifen, transcriptional regulation in response to the natural ligand of the receptor, estradiol, is disturbed.

### Acquired sensitivities and co-resistances upon development of tamoxifen resistance

**Fig. 2:**
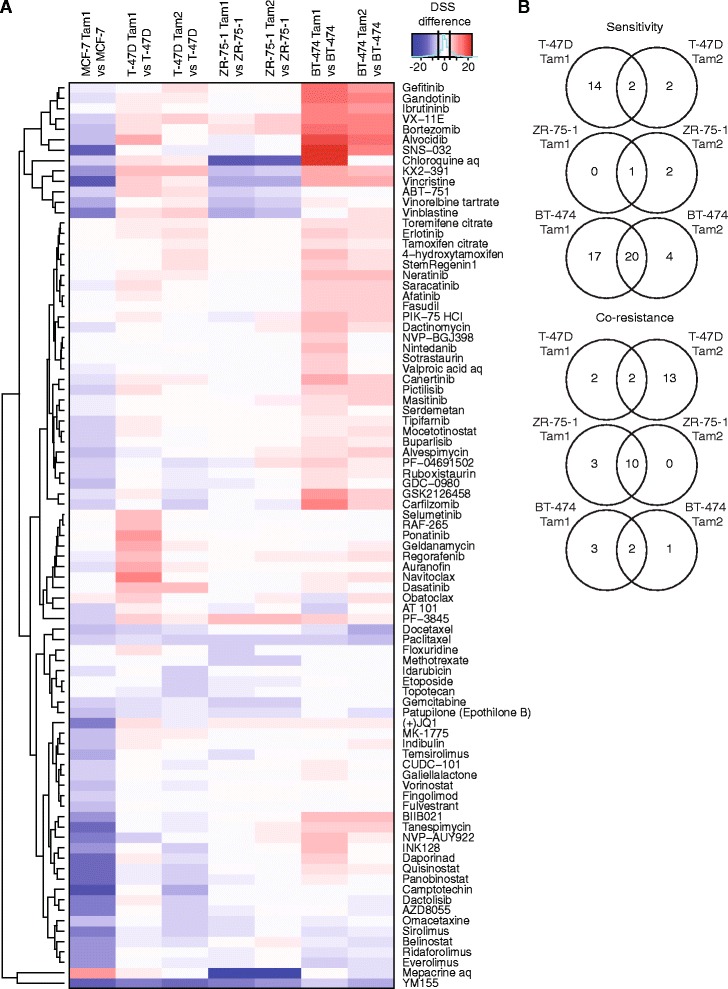
Tamoxifen-resistant cells display distinct drug response profiles. **a** Hierarchical clustering and heat map visualization of the drug sensitivity score (DSS) differences of each resistant/parental cell line pair. Red (positive DSS difference) represents sensitivity and blue (negative DSS difference) co-resistance. Drugs with DSS difference < 5 in all of the comparisons were omitted. Black lines in the color bar highlight the cutoff (5, −5). **b** Tamoxifen-resistant clones derived from same parental cells display distinct drug response profiles. Venn diagrams show overlap of drugs that the cells become sensitive or co-resistant towards. Drugs with DSS difference < 5 were omitted

To uncover drug response profiles associated with long-term tamoxifen treatment mimicking the prolonged exposure to the drug used in the clinic, we conducted drug sensitivity and resistance testing (DSRT) [26] with 279 FDA/EMA-approved and investigational oncology drugs ranging from conventional chemotherapeutics to a variety of targeted drugs (Additional files 5 and 6). We quantified the overall drug responses for each compound in each cell line using drug sensitivity score (DSS) [35]. Targeted drugs generally had higher efficacy than the standard chemotherapeutics, as seen for example with the HER2/EGFR inhibitors lapatinib, afatinib and neratinib that were efficacious only against the BT-474 parental cells that express these receptors (Additional file 2A and B). We then calculated the DSS difference between the tamoxifen-resistant and their parental cells to reveal the cell line-specific drug sensitivity and resistance patterns associated with the resistance to tamoxifen. Interestingly, all tamoxifen-resistant cell lines displayed distinct drug response profiles, with a few overlapping responses (Fig. 2a). Overall, the drug responses of the resistant clones derived from the same parentals resembled each other more than those of the other resistant clones, and a few shared sensitivities arose, such as towards tyrosine kinase (gefinitib, ibrutinib) and cdk inhibitors (alvocidib, SNS-032) in the BT-474 clones. However, in T-47D and ZR-75-1, only a few common drug sensitivities and co-resistance patterns evolved between the two subclones, indicating clonal evolution (Fig. 2b).

### Resistant cells accumulate genetic alterations throughout their genomes

To define the mutational landscape of the tamoxifen-resistant cell clones and to identify potential genomic markers for drug sensitivity and resistance, we conducted exome-sequencing on each isogenic parental-resistant cell line pair. In order to identify resistance-specific point mutations and copy number variations, each tamoxifen-resistant cell line was compared to its parental cell line. Between 31,8 and 83,0 million uniquely mapping reads per sample were obtained with an average coverage of 43,7x (Additional file 7). We found genetic changes scattered along the genomes of the tamoxifen-resistant cells (Additional files 8, 9 and 10). We identified approximately 250 to 350 non-synonymous mutations per resistant cell line (Additional file 9), and for integration with copy number alterations, selected the high confidence ones. Interestingly, only a limited panel of mutated genes was shared between the two clones derived from the same parental cells including *TIMM23* and *RP11-368 J21.2.1* in ZR-75-1 Tam1 and Tam2, and *TNS1*, *PTH2R* and *NHLRC2* in BT-474 Tam1 and Tam2. None of the point mutations were shared between T-47D Tam1 and Tam2 (Additional file 8). All resistant clones displayed marked copy number aberrations, which were primarily large deletions, with the exception of ZR-75-1 Tam1 that only harbored gains. Chromosomes X and seven were recurrently altered, with few larger deletions (such as a homo- and heterozygous deletion of Xq in MCF-7 Tam1 and heterozygous deletion of 7q in T-47D Tam1), as well as several smaller aberrations found in each resistant cell line (Additional files 8 and 10). Again, just a few genes displaying copy number alterations were shared between the two resistant clones derived from ZR-75-1 and BT-474, implying genetic clonal divergence.

### T-47D tamoxifen-resistant cells develop shared as well as individual sensitivities

**Fig. 3:**
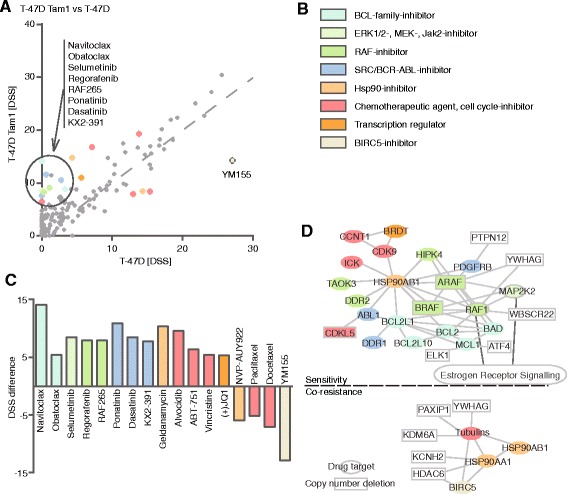
Drug testing and molecular profiling reveal sensitivity and co-resistance networks in T-47D Tam1. **a** DSS differences of tamoxifen-resistant T-47D Tam1 vs parental cells reveal emerging sensitivities (above the dotted line) and co-resistances (below the dotted line) upon acquiring tamoxifen resistance. **b** Color legend of the drug target class. For visualization purposes, the drugs were colored according to their target class as indicated, and the coloring matched with their target genes. **c** Drugs with DSS difference >5. Positive values indicate sensitivity and negative co-resistance. **d** Matching of the drugs that the cells show acquired sensitivity or co-resistance towards with their specific target genes reveals molecular networks behind sensitivity and co-resistance in T-47D Tam1. Drugs without target genes in the networks are not displayed. Drug targets (colored) and upstream molecules (uncolored) are denoted as follows: ovals, molecules without genomic changes; rectangles with solid line, molecules with copy number deletions, high confidence (*p* < 0,05 and resistant/parental frequency >30 %) point mutations could not be connected to the network and are thus not displayed. Molecules that are connected with the ER signalling pathway are connected by a dark grey line to the boxed text “Estrogen Receptor Signalling”

**Fig. 4:**
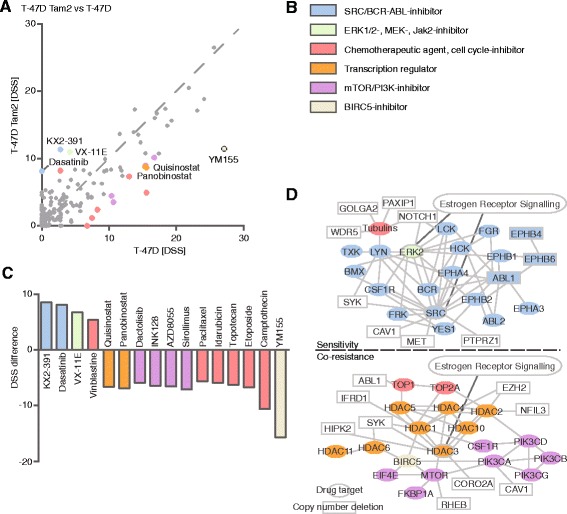
Drug testing and molecular profiling reveal sensitivity and co-resistance networks in T-47D Tam2. **a** DSS differences of tamoxifen-resistant T-47D Tam2 vs parental cells reveal emerging sensitivities (above the dotted line) and co-resistances (below the dotted line) upon acquiring tamoxifen resistance. **b** Color legend of the drug target class. For visualization purposes, the drugs were colored according to their target class as indicated, and the coloring matched with their target genes. **c** Drugs with DSS difference >5. Positive values indicate sensitivity and negative co-resistance. **d** Matching of the drugs that the cells show acquired sensitivity or co-resistance towards with their specific target genes reveals molecular networks behind sensitivity and co-resistance in T-47D Tam2. Drugs without target genes in the networks are not displayed. Drug targets (colored) and upstream molecules (uncolored) are denoted as follows: ovals, molecules without genomic changes; rectangles with solid line, molecules with copy number deletions, high confidence (*p* < 0,05 and resistant/parental frequency >30 %) point mutations could not be connected to the network and are thus not displayed. Molecules that are connected with the ER signalling pathway are connected by a dark grey line to the boxed text “Estrogen Receptor Signalling”

**Fig. 5:**
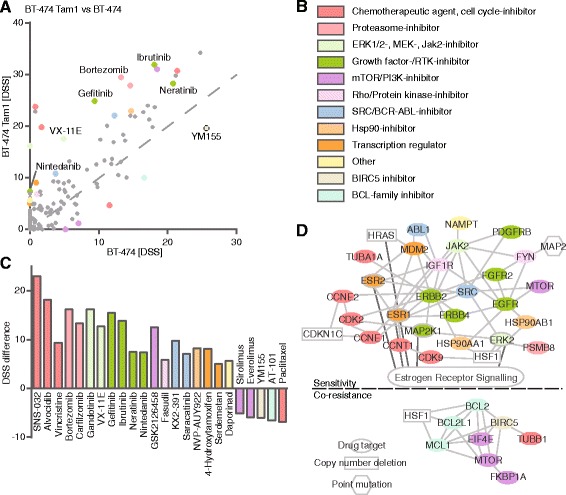
Drug testing and molecular profiling reveal sensitivity and co-resistance networks in BT-474 Tam1. **a** DSS differences of tamoxifen-resistant BT-474 Tam1 vs parental cells reveal emerging sensitivities (above the dotted line) and co-resistances (below the dotted line) upon acquiring tamoxifen resistance. **b** Color legend of the drug target class. For visualization purposes, the drugs were colored according to their target class as indicated, and the coloring matched with their target genes. **c** Drugs with DSS difference >5. Positive values indicate sensitivity and negative co-resistance. **d** Matching of the drugs that the cells show acquired sensitivity or co-resistance towards with their specific target genes reveals molecular networks behind sensitivity and co-resistance in BT-474 Tam1. Drugs without target genes in the networks are not displayed. Drug targets (colored) and upstream molecules (uncolored) are denoted as follows: ovals, molecules without genomic changes; rectangles with solid line, molecules with copy number deletion, and polygons molecules with high confidence (*p* < 0,05 and resistant/parental frequency >30 %) point mutations. Molecules that are connected with the ER signalling pathway are connected by a dark grey line to the boxed text “Estrogen Receptor Signalling”

**Fig. 6:**
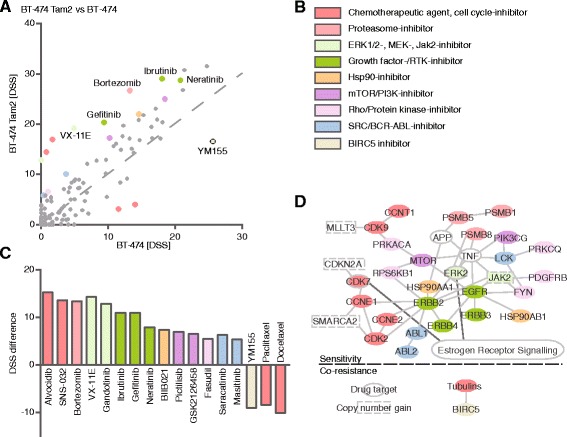
Drug testing and molecular profiling reveal sensitivity and co-resistance networks in BT-474 Tam2. **a** DSS differences of tamoxifen-resistant BT-474 Tam2 vs parental cells reveal emerging sensitivities (above the dotted line) and co-resistances (below the dotted line) upon acquiring tamoxifen resistance. **b** Color legend of the drug target class. For visualization purposes, the drugs were colored according to their target class as indicated, and the coloring matched with their target genes. **c** Drugs with DSS difference >5. Positive values indicate sensitivity and negative co-resistance. **d** Matching of the drugs that the cells show acquired sensitivity or co-resistance towards with their specific target genes reveals molecular networks behind sensitivity and co-resistance in BT-474 Tam2. Drugs without target genes in the networks are not displayed. Drug targets (colored) and upstream molecules (uncolored) are denoted as follows: ovals, molecules without genomic changes; rectangles with dashed line, molecules with copy number gain, high confidence (*p* < 0,05 and resistant/parental frequency >30 %) point mutations could not be connected to the network. Molecules that are connected with the ER signaling pathway are connected by a dark grey line to the boxed text “Estrogen Receptor Signalling”

To reveal the pharmacogenomic relationships between the drug response profiles and the genetic alterations, we integrated the drug response and genetic profiling data. Specifically, based on the DSS differences, we first selected the most pronounced drug response changes between each parental-resistant cell line pair. To correlate the genomic alterations with the drug responses we then constructed sensitivity and resistance networks among the target genes of the selected drugs, and mapped the point mutations and CNV changes onto these molecular networks as detailed in Materials and Methods, Construction of drug sensitivity and co-resistance networks. We also merged the obtained networks with the IPA canonical pathway for Estrogen Receptor Signaling. However, only in two out of fourteen networks more than two overlapping molecules were found, indicating that only a fraction of them are actually connected with the ER signaling pathway and hence, in the majority of cases, the sensitivity and resistance mechanisms arise independently of ER. Overall, the number of genetic alterations mapping onto the drug target networks varied between 11 (T-47D Tam2 sensitivity network) and 0 (some ZR-75-1 and BT-474 networks), and converged into a number of target genes. In all but two networks (BT-474 Tam2 and ZR-75-1 Tam2 sensitivity) all the observed CNV changes were heterozygous deletions (Figs. 3, 4, 5 and 6 and Additional files 11 and 12). Of the two tamoxifen-resistant clones generated from the parental T-47D, both exhibited mostly sensitized profiles. T-47D Tam1 and Tam2 both acquired sensitivity towards the SRC/ABL-family inhibitors; the Tam1 cells towards three of them: ponatinib, dasatinib and KX2-391; and the Tam2 cells against two of them: dasatinib and KX2-391 (Figs. 3 and 4). The Tam1 cells also became sensitive towards selumetinib, a MEK inhibitor, and the Tam2 cells towards VX-11E, an ERK1/2 inhibitor. In addition, sensitivity towards BCL-family inhibitors (navitoclax, obatoclax), and RAF inhibitors (regorafenib, RAF265) was observed in Tam1, but not in Tam2. Reflecting the acquired drug sensitivities, multiple target genes of these drugs or their upstream effectors converged into the same sensitivity networks, and notably, many of these target genes also harbored genetic changes.

### Increased sensitivity towards HER2/EGFR inhibitors

Similar to the T-47D resistant clones, the tamoxifen-resistant BT-474 s displayed mainly acquisition of drug sensitivities. However, unlike any of the other tamoxifen-resistant clones, BT-474 Tam1 and Tam2 developed vulnerability to several EGFR/HER2-inhibitors (gefitinib, ibrutinib, neratinib, nintedanib; Fig. 5a, c and Fig. 6a, c). Notably, the parental BT-474 s also showed sensitivity towards HER2/EGFR inhibitors (Additional file 2), which was selectively enhanced upon developing resistance to tamoxifen. In addition, sensitivity towards cdk-inhibitors (SNS-032, alvocidib) emerged, and several of the drugs’ target genes or their upstream effectors also harbored CNV changes (Fig. 6d). Both tamoxifen-resistant BT-474 clones also developed sensitivity towards the ERK1/2-inhibitor VX-11E and the JAK2-inhibitor gandotinib. BT-474 Tam2 also displayed gain of *JAK2*, possibly explaining the developing sensitivity towards gandotinib (Fig. 6d).

### Co-resistance against multiple drugs evolves across the tamoxifen-resistant cells

In addition to emerging drug sensitivities, we also observed acquired co-resistances upon development of tamoxifen resistance. In contrast to the other resistant cells that developed both new sensitivities as well as co-resistances, the tamoxifen-resistant MCF-7 Tam1 displayed an overwhelmingly co-resistant drug response profile, including resistance towards many chemotherapeutics (camptothecin, vincristine, SNS-032), but also several mTOR- and two HDAC-inhibitors (dactolisib, AZD8055, sirolimus, panobinostat, belinostat) (Additional file 11). In the two T47-D tamoxifen clones, the resistance networks were markedly different, with T47-D Tam1 displaying resistance merely to four agents, two of which were chemotherapeutics, whereas T-47D Tam2 cells exhibited co-resistance to a large variety of drugs. These included some of the same drugs as for the Tam1 clone, and additionally Tam2 showed resistance to four mTOR- and two HDAC-inhibitors (Figs. 3 and 4). Both ZR-75-1 as well as the BT-474 resistant clones developed co-resistance to several chemotherapeutics (Figs. 5 and 6, Additional files 5, 6, 12), with the ZR-75-1 networks being nearly identical, reflecting the high similarity between their drug response patterns. Interestingly, the BT-474 Tam1 cells additionally showed resistance to rapamycin and everolimus, two mTOR inhibitors, as well as to AT 101, a BCL-family inhibitor (Fig. 5). Collectively, compared to the sensitivity profiles, the resistance networks demonstrated less variance between the different tamoxifen-resistant clones, with the majority of them developing co-resistance towards common chemotherapeutics (Table 1).

**Table 1:** Tamoxifen-resistant cells develop individual as well as common drug sensitivities and co-resistances. Sensitizing and desensitizing drugs, drug target classes, specific target genes and as well as affected cell lines are listed

Sensitivity towards	Drug target class	Specific target gene	Observed in cell line
Navitoclax, Obatoclax	BCL-family	BCL2L1	T-47D Tam1
		BAD	
RAF265, Ponatinib	RAF-family	RAF1	T-47D Tam1
Dasatinib, KX2-391	SRC/ABL	ABL1	T-47D Tam1 & Tam2
		SRC	
VX-11E	MAPK1	MAPK1	T-47D Tam2, BT-474 Tam2, ZR-75-1 Tam2
Gefitinib, Ibrutinib, Neratinib, Nintedanib	HER2/EGFR	ERBB2	BT-474 Tam1 & Tam2
		ERBB4	
		ERBB3	
Vinblastine	Tubulins	TUBA1A	T-47D Tam2
		TUBA4A	
		TUBA1C	
		TUBB6	
PF-3845	FAAH	FAAH	ZR-75-1 Tam2
Bortezomib	PSMB-family	PSMB5	ZR-75-1 Tam2
Resistance towards	Drug target class	Specific target gene	Observed in cell line
YM155	BIRC5	BIRC5	T-47D Tam1 & Tam2, MCF-7 Tam1, BT-474 & Tam2
Quisinostat, Panobinostat	HDACs	HDAC1	T-47D Tam2, MCF-7 Tam1
		HDAC6	
Docetaxel, Paclitaxel	Tubulins	TUBA1C	BT-474 Tam2
		TUBB2A	
		TUBB3	
		TUBB	
		TUBB4B	
		TUBB6	
Temsirolimus, Everolimus, Ridaforolimus, INK128, Sirolimus, AZD8055, Dactolisib	mTOR	mTOR	MCF-7 Tam1

### Shared sensitivities and co-resistances

**Fig. 7:**
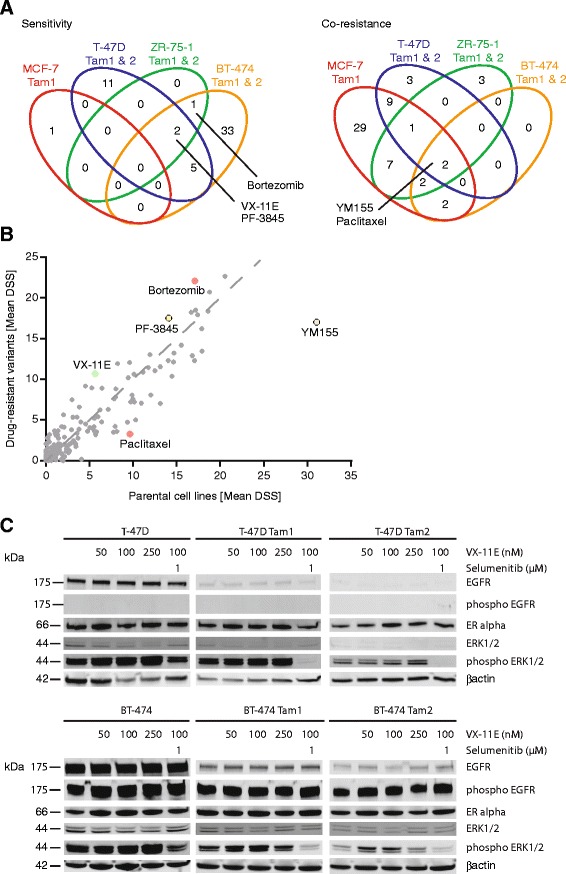
Tamoxifen-resistant cells develop common drug sensitivities and co-resistances. **a** Venn diagrams illustrate shared drug sensitivities and co-resistances of drugs with a DSS difference of at least 5. **b** Rank-product analysis of Drug Sensitivity Scores (DSS) between tamoxifen-resistant and parental cell lines identified statistically significant shared sensitizing and desensitizing drugs. **c** Western blotting of EGFR, pEGFR, ERK1/2, pERK1/2, ERα and βactin under increasing concentrations of VX-11E and 1 μM selumetinib of T-47D and BT-474 isogenic cell lines. Resistant cell lines were cultured in media supplemented with 1 μM tamoxifen

We next addressed the development of common sensitivities and co-resistances upon acquisition of tamoxifen resistance. Cross-comparison of the drug response profiles across all tamoxifen-resistant clones revealed that several of them developed sensitivity towards the ERK1/2-inhibitor VX-11E, the proteasome-inhibitor bortezomib, and the FAAH-inhibitor PF-3845. Common co-resistance towards the survivin-inhibitor YM155 and the chemotherapeutic agent paclitaxel also occurred (Fig. 7a). Furthermore, even with the limited sample size, these shared responses were statistically significant (rank product analysis, Fig. 7b). To further assess the EGFR/ERK signaling pathway in the resistant cells, we selected the MEK/ERK inhibitors for which a differential drug sensitivity score was obtained, i.e. VX-11E and selumetinib. The concentration range was selected based on the IC_50_ of the drugs in the individual cell lines (Additional file 5). We then cultured the cells either in their default culture media, or with increasing concentrations of VX-11E, or with VX-11E and selumetinib concomitantly, and performed Western blotting with ERK1/2 and EGFR antibodies (Fig. 7c). The basal levels of these unphosphorylated as well as phosphorylated signaling proteins were lowered in the tamoxifen-resistant cells compared to the parentals (Fig. 7c), but upon increasing concentrations of VX-11E, slight increase in phosphorylated ERK1/2 was observed in the T-47Ds. However, upon additional inhibition of MEK with selumetinib, the increase in phosphorylated ERK1/2 was diminished.

## Discussion

In the present study, we report the development and systematic characterization of seven long-term tamoxifen-treated cell lines, and by pharmacogenomic profiling, determine the drug response profiles and mutational landscapes of these drug-resistant models. Different *in vitro* and *in vivo* models of endocrine-resistance have been developed to explore common mechanisms behind resistance development [9–11, 19, 20, 22, 40–50] However, to our knowledge, this is the first comprehensive drug testing study with hundreds of oncology compounds across a panel of several tamoxifen-resistant models. Using this approach, we identify clone-specific molecular networks reflecting the diversity of pathways leading to endocrine resistance. It is noteworthy that as the availability of clinical data sets on diagnosed acquired tamoxifen resistance with response/survival data are essentially non-existent to date, the resistant/sensitive cell line models and associated data sets presented here form a valuable research resource.

Concurrently with developing tamoxifen resistance, novel drug vulnerabilities emerge. Here, we identified common, cell type-, and cell clone-specific sensitivities. The most important of these are listed in Table 1. Additionally, several of the sensitizing drugs are in clinical trials for treatment of advanced or metastatic breast cancer. However, possible correlation between patient enrollment criteria, observed molecular mechanisms and the sensitivities and co-resistances identified here remains to be investigated.

All resistant cell lines except one (MCF-7 Tam1) exhibited gained sensitivity towards the ERK1/2 inhibitor VX-11E. ERK1/2 inhibition prevents its autophosphorylation [10, 51] and results in reduced phosphorylation and thereby also decreased activation of ER [9]. Overactivity of ERK1/2 has been shown to associate with loss of ER, and decreased levels of ER are also seen in the majority of our tamoxifen-resistant cell lines (Additional file 4) [52]. However, the basal levels of unphosphorylated or phosphorylated EGFR/ERK are not elevated in the tamoxifen resistant lines; rather the opposite, i.e. decrease in basal levels as well as dephosphorylation of ERK1/2 and EGFR is observed (Fig. 7c and data not shown). We therefore anticipate that increased phosphorylation of these signaling proteins does not explain the observed sensitivity towards VX-11E. Interestingly, upon increasing concentrations of VX-11E, slight increase in phosphorylated ERK1/2 is observed in the T-47D Tam clones. However, upon additional inhibition of MEK with selumetinib, this effect is diminished. The effect of VX-11E inducing ERK1/2 phosphorylation has also previously been reported [53] and might therefore reflect a general mode of action especially as the same effect is observed also in our parental cells (Fig. 7c). It could therefore be speculated that already a short-term tamoxifen treatment causes an effect on the levels of phosphorylated ERK1/2 and that long-term exposure leads to, at least partial, down-regulation of EGFR and ERK1/2. As cells are challenged with increasing concentrations of an ERK1/2 inhibitor (VX-11E), an increase in ERK1/2 phosphorylation is seen, with concomitant cell killing of the tamoxifen-resistant cells observed in the drug screen. However, further studies to elucidate the exact mechanisms are needed.

We also identified bortezomib, a proteasome inhibitor, as a sensitizing agent (Figs. 5, 6 and 7 and Additional file 12, Table 1). A direct role for bortezomib in reversing tamoxifen resistance has not been demonstrated before, although a link between proteasome function and estrogen receptor -mediated transcription has been suggested [54], and bortezomib has recently been shown to enhance endocrine treatment in cell line models as well as in humans [11–13].

In addition to shared sensitivity to VX-11E and bortezomib in the tamoxifen-resistant cells, we also identified cell line specific drug sensitivities (Table 1, Figs. 3, 4, 5 and 6, Additional files 11 and 12). T-47D Tam1 and Tam2 cells displayed sensitivity towards the SRC-family inhibitor KX2-391 and the dual ABL/SRC-inhibitors dasatinib and ponatinib (Figs. 3 and 4). This is in agreement with recent findings [20, 55]. Another SRC-inhibitor, SU6656, has also been reported to inhibit growth of tamoxifen-resistant cells [42], highlighting the potential of SRC-inhibition in overcoming endocrine resistance. Interestingly, dasatinib has been shown to overcome tamoxifen resistance in MCF-7/fibroblast co-culture, and it is currently undergoing clinical trials on metastasized ER-positive breast cancer.

Upon acquiring resistance to tamoxifen, the T-47D Tam1 cells also gained sensitivity towards the BCL-family inhibitors navitoclax and obatoclax (Fig. 3). BCL-2 family proteins BCL-2, BCL2L1, BCL2L10 and MCL1, represented in the network, are major negative regulators of apoptosis, and thus, upregulation of their expression might offer the tamoxifen-challenged cells means to overcome resistance, as well as downregulation of BAD, a proapoptotic regulator. Indeed, BCL-2 has been indicated in tamoxifen resistance, and consequently, a BCL-2 inhibitor, ABT-737, has been reported to restore sensitivity [56]. Additionally, tamoxifen treated patients with low level of BAD expression had a worse prognosis [57]. The T-47D Tam1 cells also displayed sensitivity towards RAF-inhibitors BAY 73–4506 and RAF265. This is in line with previous findings on overexpression of *RAF1* promoting tamoxifen-resistant growth [58]. Both navitoclax and BAY 73–4506 are being investigated for treatment of different cancers, navitoclax for lung cancer and lymphoma, and BAY 73–4506 for metastatic colorectal cancer among others. Our results, and those from others [56–58] suggest that BCL- and RAF-inhibitors might offer means to treat also endocrine-resistant breast cancer.

We also identified sensitizers with preference for the luminal B-derived tamoxifen-resistant cells, BT-474 Tam1 and Tam2. These included the cdk-inhibitors SNS-032 and alvocidib, along with the EGFR-inhibitor gefinitib and the Btk-inhibitor PCI-32765, and several HER2/EGFR-inhibitors (Figs. 5 and 6). Crosstalk between ER and ERBB2/EGFR pathways has been shown to be activated in tamoxifen resistance [59]. Recently, the EGFR/HER2 dual inhibitor AZD8931 was also suggested to inhibit growth of MCF-7 or T-47D tamoxifen-resistant cells in xenograft models [60]. It is noteworthy that in our study, the parental BT-474 cells, unlike all others presented here, initially amplify and overexpress HER2, and display some sensitivity towards HER2/EGFR-inhibitors (Additional file 2). Interestingly, as tamoxifen resistance develops, the cells become more sensitive to several of the HER2/EGFR-inhibitors and indeed, the combined use of growth factor receptor kinase inhibitors in conjunction with tamoxifen has been suggested to circumvent endocrine resistance [45], and combination therapy with antihormone and gefinitib has demonstrated resensitization to tamoxifen in xenografts [61, 62]. However, our results on decreasing EGFR / phospho-EGFR levels upon acquired resistance (Fig. 7c) indicate that mechanisms other than direct upregulation of the EGFR pathway are responsible for the observed gained sensitivity.

Development of primary drug resistance in cancer treatment frequently results in the emergence of secondary resistances. Here, we discovered that upon acquiring tamoxifen resistance, all of the resistant cells acquired co-resistance towards at least one chemotherapeutic agent, such as paclitaxel, docetaxel, vincristine, vinblastine or topotecan (Figs. 2, 3, 4, 5, 6 and 7, Table 1 and Additional files 5, 11 and 12). Even though chemoresistance has been associated with the estrogen receptor [24], the co-resistance observed here may rather reflect the slowed-down growth of many of the resistance clones, and may therefore propose a uniform mechanism for paclitaxel resistance (Additional file 3). However, selective co-resistance still occurs, as the cells do not become universally co-resistant against all chemotherapeutics. Nevertheless, general down-regulation of cellular functions is especially evident with the tamoxifen-resistant MCF-7 Tam1 cells, which not only possess an overwhelmingly co-resistant drug response profile, but also down-regulate cell signaling (Additional files 3, 4, 11). Indeed, already a short-term tamoxifen-treatment of MCF-7 cells triggers a predominant down-regulation of gene expression [63], suggesting that depending on the molecular background, some tamoxifen-resistant cells might exhibit an intrinsically more unresponsive profile.

Interestingly, every single tamoxifen-resistant cell line was also more resistant to the survivin (BIRC5-) inhibitor YM155 than their parental counterparts (Figs. 2, 3, 4, 5, 6 and 7, Table 1 and Additional files 5, 11 and 12), suggesting a role for survivin in development of tamoxifen resistance. Survivin has recently been associated with resistance to chemo- or hormonal therapy, and has been identified to predict poor clinical outcome via ERBB2-mediated overexpression [47]. Furthermore, siRNA-knock down of *BIRC5* has been shown to enhance cell sensitivity to tamoxifen [64]. Alternatively, it has been speculated that uptake of YM155 is dependent on cell membrane a solute carriers, encoded by the *SLC35F2* gene [65]. Upon resistance development, expression of the solute carriers possibly decreases and consequently, less YM155 enters the cells making them resistant to the drug.

As initiation and development of acquired tamoxifen resistance are largely thought to be driven by genetic adaptations [3–5, 7] we inspected the genetic landscape of the drug-resistant cells by exome-sequencing and correlated the findings with our drug profiling data. Tamoxifen-resistant cells accumulated point mutations and copy number changes throughout their genomes, with only some of the changes being common between two resistant clones originating from the same parental cells, implying clonal divergence (Additional files 8, 9 and 10). Whilst many of the genetic aberrations that have been associated with endocrine resistance previously were also recapitulated here, our data as a whole demonstrate that new sensitivities may develop largely independent of the genetic changes, and in fact, antiestrogen resistance can be seen even in the absence of any evident mutations [66]. Analogous phenomenon has been noted in leukemic cells [67]. Accumulation of numerous genomic aberrations can trigger resistance development [20], but mutations can also be carried along as passengers as a result of selection pressure, rather than emerge as true evolutionary drivers [68]. The data presented here demonstrate that in the majority of cases, no single genetic alteration can be identified as responsible for the drug response, but on the contrary, multiple target genes of the drugs converge into the same response networks, and many of these target genes also harbor genetic changes. Therefore, resistance development is likely to involve complex interactions comprising genetic as well as transcriptional and epigenetic mechanisms, or other adaptive changes in cell signaling.

## Conclusion

Taken together, the results presented in this study demonstrate that upon acquiring endocrine-resistance, breast cancer cells follow different paths to resistance, as shown by distinct genomic evolution. As a consequence, gained sensitivity as well as co-resistance towards a variety of other agents evolves. In addition to common vulnerability towards ERK1/2- and proteasome-inhibitors, we also identified a universal co-resistance towards the survivin-inhibitor YM155 in tamoxifen-resistant cells. Drug response profiles between cell clones derived from the same parental cells differed markedly, and different members of the same drug classes could be either sensitizing or desensitizing. This suggests that resistance mechanisms vary within tumors, among patients and with time, highlighting the need for personalized diagnosis and clone-targeting therapies in the treatment of tamoxifen-resistant breast cancer. As shown here, prediction of drug responses can be difficult based on genomic profiling alone. This study provides a reference set of materials (drug-resistant cell lines), and their cell biological, genomic and drug response profiling for future studies aiming to test novel therapies for breast cancer with acquired tamoxifen resistance.

## Abbreviations

ER, estrogen receptor; DSRT, drug sensitivity and resistance testing; DSS, drug sensitivity score; KIBA–score, kinase inhibitor bioactivity –score; Ki, inhibitory constant; Kd, dissociation constant; IC50, half maximal inhibitory concentration; IPA, ingenuity pathway analysis; CNV, copy number variation; cdk, cyclin-dependent kinases

## Additional files

Additional file 1: Primer sequences. (XLSX 10 kb) Additional file 2: Parental cells show sensitivity and selectivity towards known breast cancer drugs according to their subtype. (A) Drug Sensitivity Scores (DSS) of known breast cancer treatment drugs. (B) DSS of drugs specific to luminal A-(left) and luminal B-subtypes (right). Results are extracted from the data on all drugs tested in all cell lines, presented in Additional file 5. The drugs specific to parental luminal A and B subtypes were identified based on their DSS scores. (PDF 423 kb) Additional file 3: Growth and tamoxifen-tolerance of tamoxifen-resistant cells. (A) CTG-viability measurement of tamoxifen-resistant vs parental cells treated with increasing concentrations of tamoxifen. (B) Measurement of active DNA synthesis by FACS-analysis showing accumulation of resistant cells in the G0/G1 phase of the cell cycle. (PDF 1035 kb) Additional file 4: Estrogen responsivity of ERα-mediated transcription in the tamoxifen-resistant cells. (A) Quantitative RT-PCR showing decreased ERα target gene expression in the resistant cells. (B) Quantitative RT-PCR of the ERα target gene pS2 expression upon estradiol withdrawal and subsequent addition of estradiol back to the cells. (C) Western blotting displaying altered protein levels of ERα in the resistant cells. A.u. = arbitrary units, E2 = 17β-estradiol. Error bars show standard deviation. (PDF 704 kb) Additional file 5: Drug Sensitivity Scores. Drugs used in the study, their approval status and Drug Sensitivity Scores (DSS) of the tamoxifen-resistant and their parental cell lines. Max.Conc [nM] = maximum concentration, Min.Conc [nM] = minimum concentration, MAX = maximum % inhibition, D1 [% inhibition]… D5 [% inhibition] = % inhibition from the lowest (D1) to the highest drug concentration (D5). (XLSX 478 kb) Additional file 6: DSRT statistics. (XLSX 9 kb) Additional file 7: Exome-sequencing reads. (XLSX 10 kb) Additional file 8: Copy number alterations and point mutations are scattered throughout the genomes of the tamoxifen-resistant cells. (A) Relative copy number of each resistant cell line measured by exome-sequencing and plotted as log2 ratio of resistant vs parental cell line (colored dots). Copy number gains/amplifications and losses/deletions are visible as peaks and valleys in the red segmentation line, respectively. Chromosomes are numbered and highlighted in alternating colors. High confidence point mutations (*p* < 0,05 and resistant/parental frequency >30 %) are depicted above the segmentation line. (B) Venn diagrams show overlap of point mutations (black) and genes altered by CNVs (red) between the tamoxifen-resistant clones derived from same parental cells. (PDF 735 kb) Additional file 9: Point mutations from exome-sequencing. Point mutations, sequencing reads and mutation frequency in tamoxifen-resistant cells (Resistant Reference Reads, Resistant Variant Reads, Resistant Variant Frequency) compared to their parental cell lines (Parental Reference Reads, Parental Variant Reads, Parental Variant Frequency) revealed by exome-sequencing. Sample ID, chromosome, position, reference and variant base, affected gene, effect, effect impact, and *p*-values are also indicated. (XLSX 250 kb) Additional file 10: Copy number variations from exome-sequencing. Copy number changes in tamoxifen-resistant cells compared to their parental cell lines as revealed by exome-sequencing. Ensembl gene IDs, Hgnc symbols, chromosomal position (Chr, start, end), copy number value (copy_num), copy number status (cn_status) and potential presence at a breakpoint are indicated. (XLSX 8980 kb) Additional file 11: Drug testing and molecular profiling reveal sensitivity and co-resistance networks in MCF-7 Tam1. (A) DSS differences of tamoxifen-resistant MCF-7Tam1 vs parental cells reveal emerging sensitivities (above the dotted line) and co-resistances (below the dotted line) upon acquiring tamoxifen resistance. (B) Color legend of the drug target class. For visualization purposes, the drugs were colored according to their target class as indicated, and the coloring matched with their target genes. (C) Drugs with DSS difference >5. Positive values indicate sensitivity and negative co-resistance. (D) Matching of the drugs that the cells show acquired sensitivity or co-resistance towards with their specific target genes reveals molecular networks behind sensitivity and co-resistance in MCF-7 Tam1. Drugs without target genes in the networks are not displayed. Drug targets (colored) and upstream molecules (uncolored) are denoted as follows: ovals, molecules without genomic changes; rectangles with solid line, molecules with copy number deletions, high confidence (*p* < 0,05 and resistant/parental frequency >30 %) point mutations could not be connected to the network and are thus not displayed. Molecules that are connected with the ER signalling pathway are connected by a dark grey line to the boxed text “Estrogen Receptor Signalling”. (PDF 443 kb) Additional file 12: Drug testing and molecular profiling reveal sensitivity and co-resistance networks in ZR-75-1 Tam1 and Tam2. (left) DSS differences of tamoxifen-resistant (A) ZR-75-1 Tam1 and (B) ZR-75-1 Tam2 vs parental cells reveal emerging sensitivities (above the dotted line) and co-resistances (below the dotted line) upon acquiring tamoxifen resistance. (middle) Drugs with DSS difference >5. Positive values indicate sensitivity and negative co-resistance. (right) Matching of the drugs that the cells show acquired sensitivity or co-resistance towards with their specific target genes reveals molecular networks behind sensitivity and co-resistance in resistant cells. Drugs without target genes in the networks are not displayed. Drug targets (colored) and upstream molecules (uncolored) are denoted as follows: ovals, molecules without genomic changes; rectangles with dashed line, molecules with copy number gain, high confidence (*p* < 0,05 and resistant/parental frequency >30 %) point mutations could not be connected to the network and are thus not displayed. (B) Color legend of the drug target class. For visualization purposes, the drugs were colored according to their target class as indicated, and the coloring matched with their target genes. Molecules that are connected with the ER signalling pathway are connected by a dark grey line to the boxed text “Estrogen Receptor Signalling”. (PDF 464 kb)
