# Supplementary figures and images for: Systematic drug screening reveals specific vulnerabilities and co-resistance patterns in endocrine-resistant breast cancer
Source: BMC Cancer. 2016 Jul 4;16:378. doi: 10.1186/s12885-016-2452-5 (PMC4932681; doi:10.1186/s12885-016-2452-5)

**A**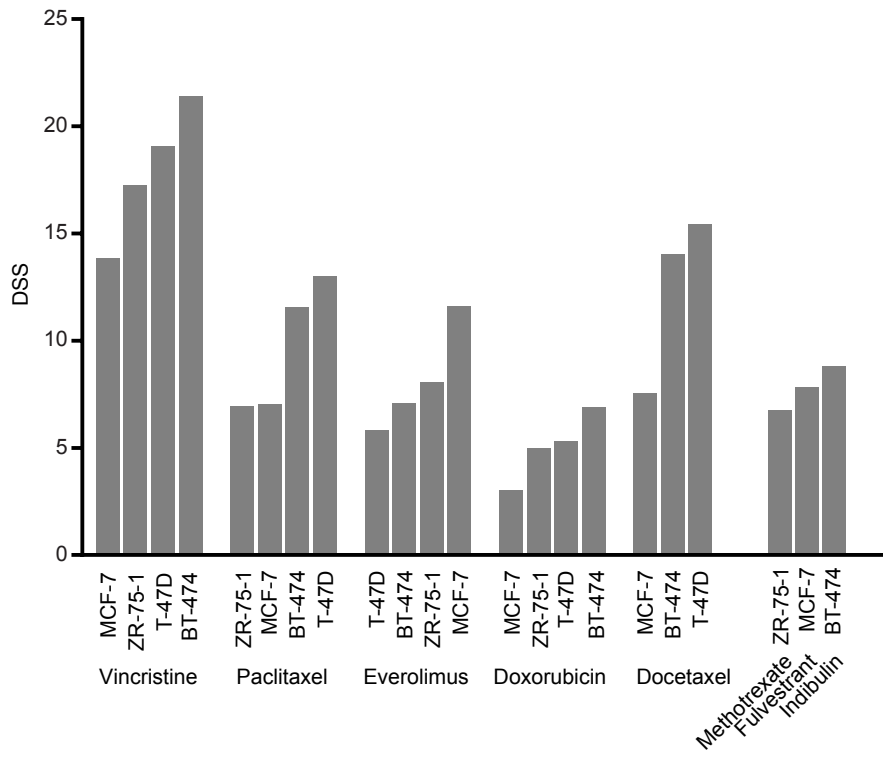**B**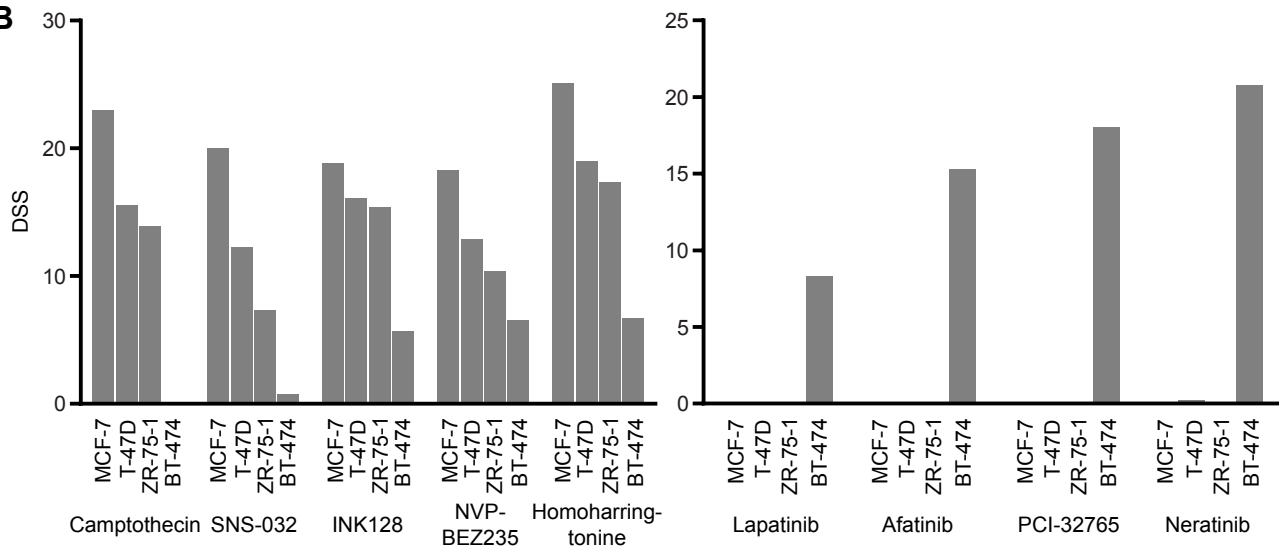

Supplement: Additional file 2: — Parental cells show sensitivity and selectivity towards known breast cancer drugs according to their subtype. (A) Drug Sensitivity Scores (DSS) of known breast cancer treatment drugs. (B) DSS of drugs specific to luminal A-(left) and luminal B-subtypes (right). Results are extracted from the data on all drugs tested in all cell lines, presented in Additional file 5. The drugs specific to parental luminal A and B subtypes were identified based on their DSS scores. (PDF 423 kb) [file 12885_2016_2452_MOESM2_ESM.pdf]

**A**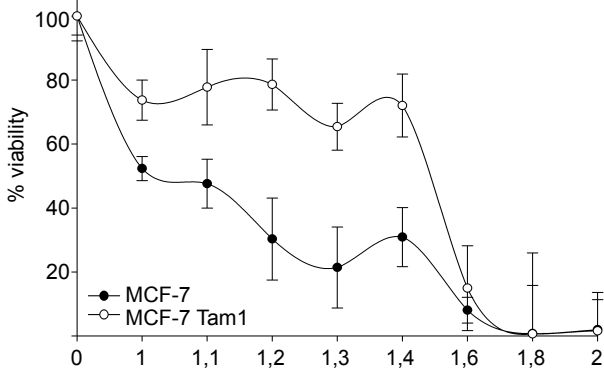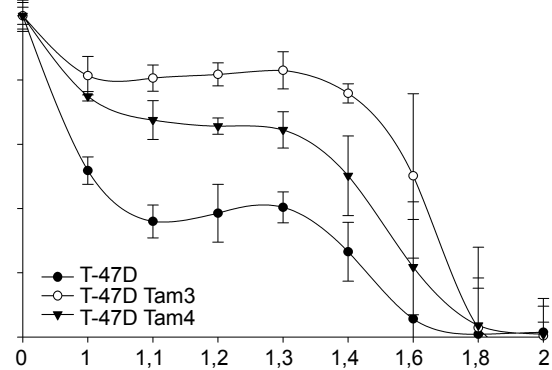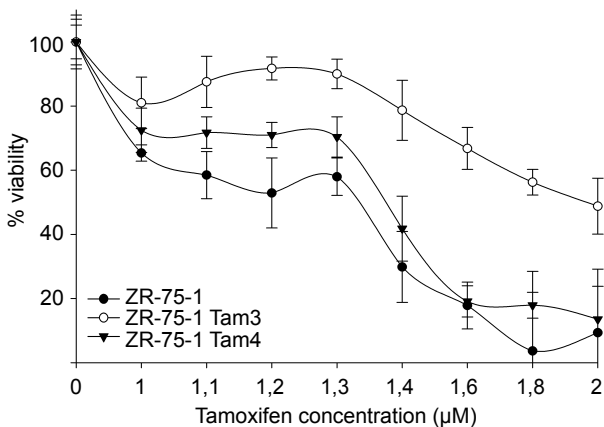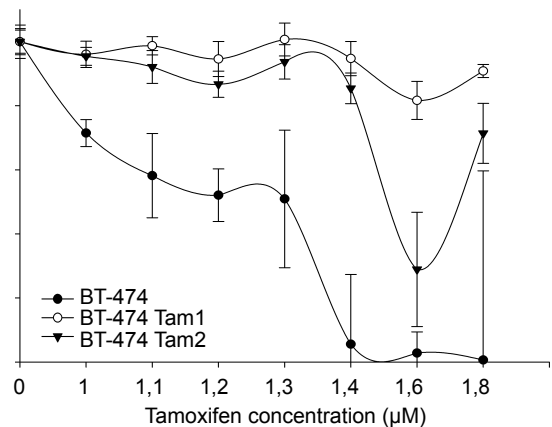**B**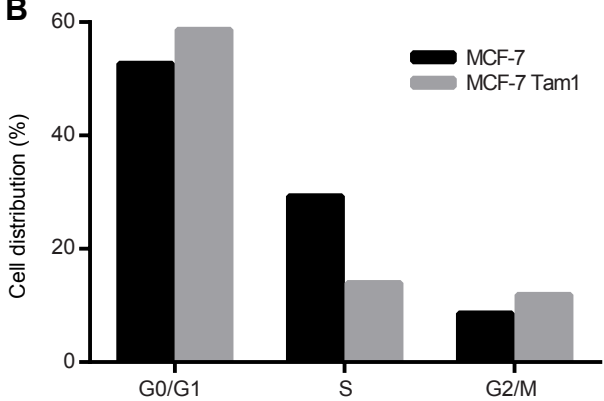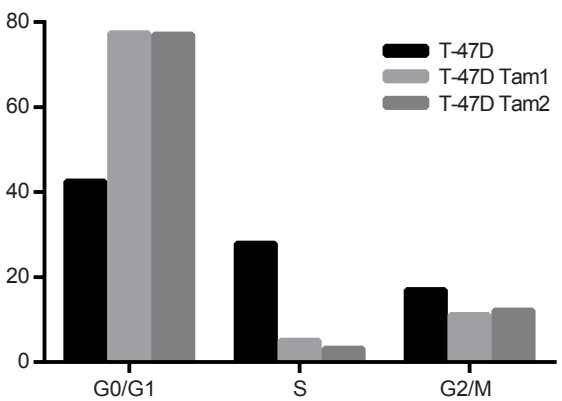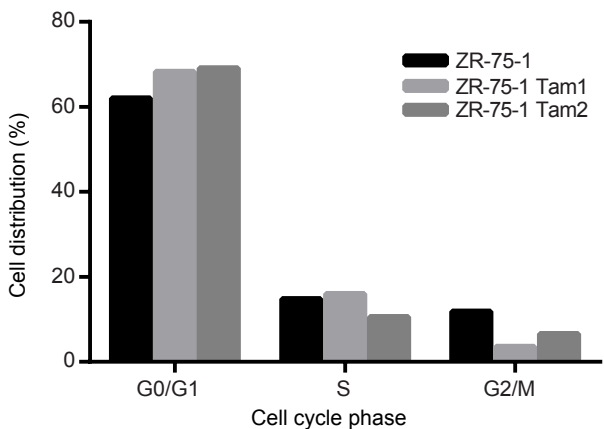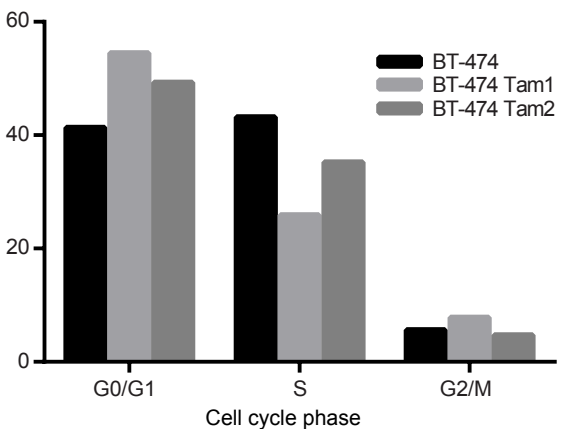

Supplement: Additional file 3: — Growth and tamoxifen-tolerance of tamoxifen-resistant cells. (A) CTG-viability measurement of tamoxifen-resistant vs parental cells treated with increasing concentrations of tamoxifen. (B) Measurement of active DNA synthesis by FACS-analysis showing accumulation of resistant cells in the G0/G1 phase of the cell cycle. (PDF 1035 kb) [file 12885_2016_2452_MOESM3_ESM.pdf]

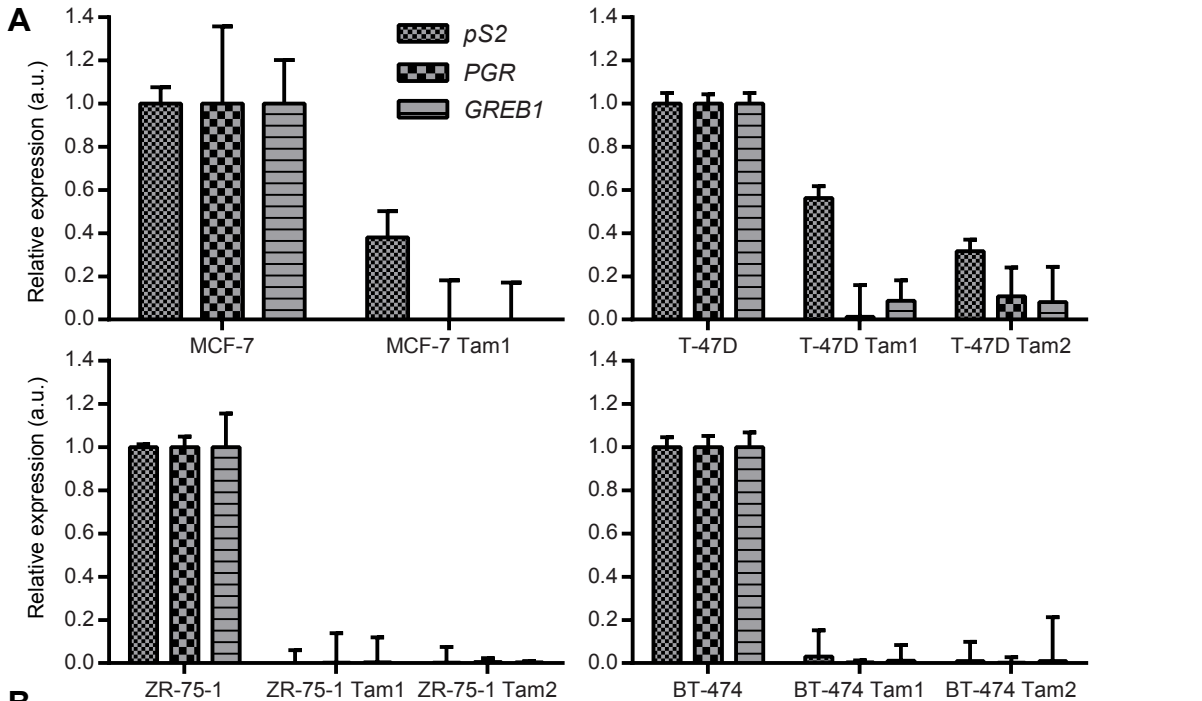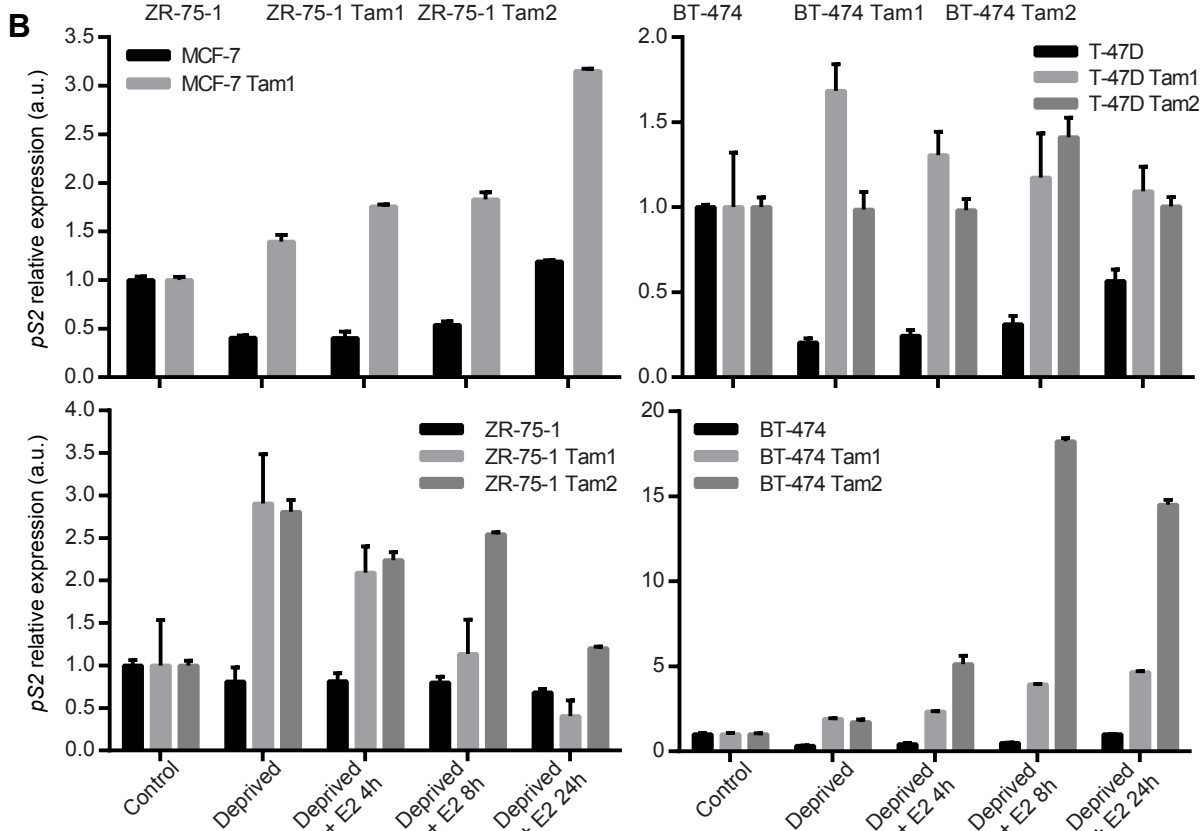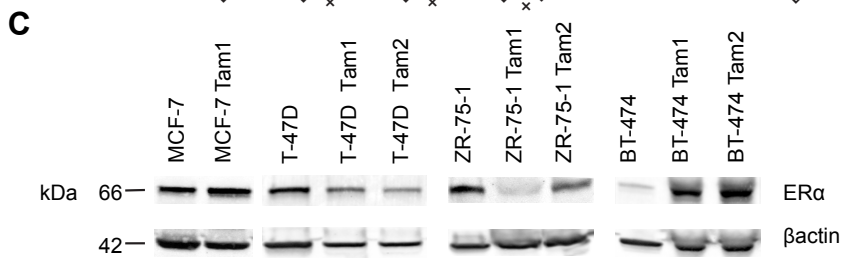

Supplement: Additional file 4: — Estrogen responsivity of ERα-mediated transcription in the tamoxifen-resistant cells. (A) Quantitative RT-PCR showing decreased ERα target gene expression in the resistant cells. (B) Quantitative RT-PCR of the ERα target gene pS2 expression upon estradiol withdrawal and subsequent addition of estradiol back to the cells. (C) Western blotting displaying altered protein levels of ERα in the resistant cells. A.u. = arbitrary units, E2 = 17β-estradiol. Error bars show standard deviation. (PDF 704 kb) [file 12885_2016_2452_MOESM4_ESM.pdf]

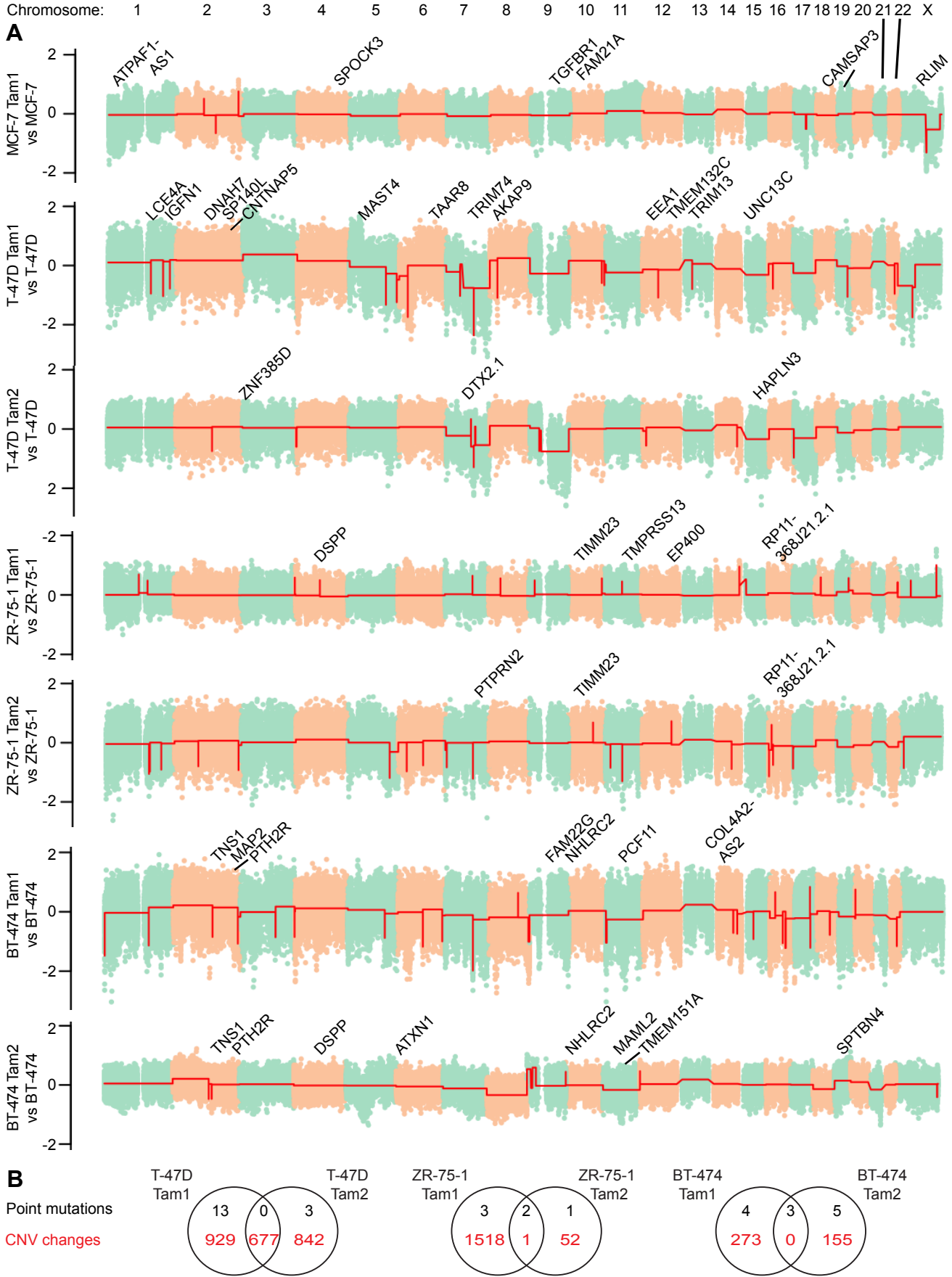

Supplement: Additional file 8: — Copy number alterations and point mutations are scattered throughout the genomes of the tamoxifen-resistant cells. (A) Relative copy number of each resistant cell line measured by exome-sequencing and plotted as log2 ratio of resistant vs parental cell line (colored dots). Copy number gains/amplifications and losses/deletions are visible as peaks and valleys in the red segmentation line, respectively. Chromosomes are numbered and highlighted in alternating colors. High confidence point mutations (p < 0,05 and resistant/parental frequency >30 %) are depicted above the segmentation line. (B) Venn diagrams show overlap of point mutations (black) and genes altered by CNVs (red) between the tamoxifen-resistant clones derived from same parental cells. (PDF 735 kb) [file 12885_2016_2452_MOESM8_ESM.pdf]

**A** MCF-7 Tam1 vs MCF-7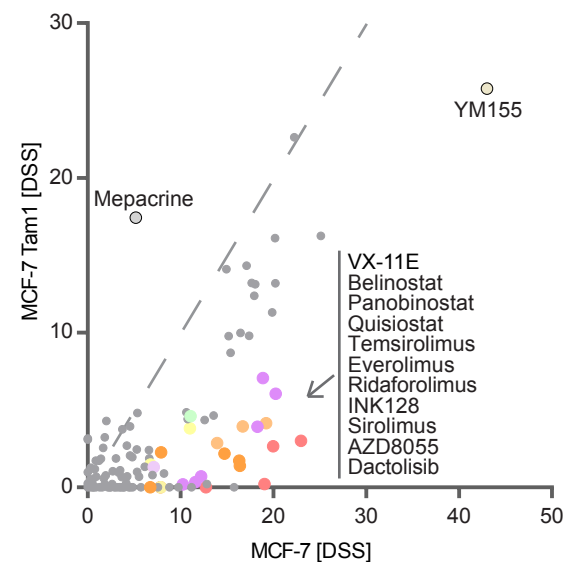**B**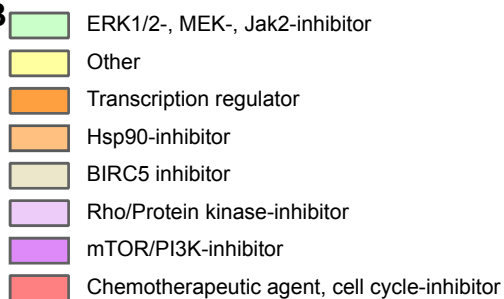**C**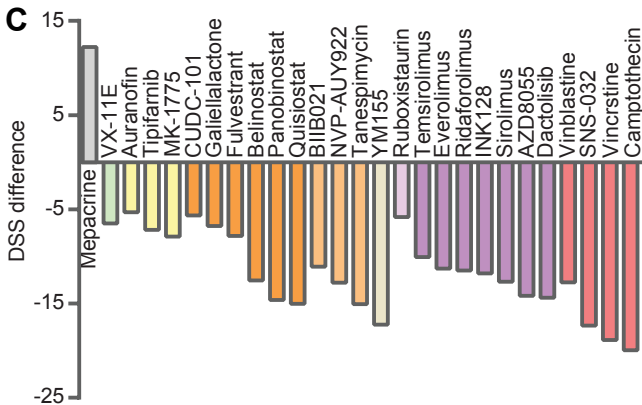**D**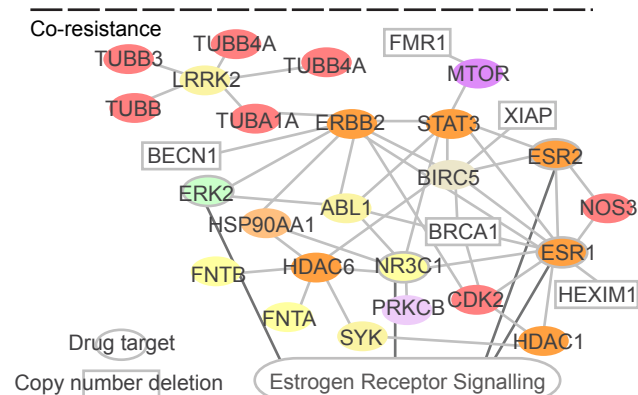

Supplement: Additional file 11: — Drug testing and molecular profiling reveal sensitivity and co-resistance networks in MCF-7 Tam1. (A) DSS differences of tamoxifen-resistant MCF-7Tam1 vs parental cells reveal emerging sensitivities (above the dotted line) and co-resistances (below the dotted line) upon acquiring tamoxifen resistance. (B) Color legend of the drug target class. For visualization purposes, the drugs were colored according to their target class as indicated, and the coloring matched with their target genes. (C) Drugs with DSS difference >5. Positive values indicate sensitivity and negative co-resistance. (D) Matching of the drugs that the cells show acquired sensitivity or co-resistance towards with their specific target genes reveals molecular networks behind sensitivity and co-resistance in MCF-7 Tam1. Drugs without target genes in the networks are not displayed. Drug targets (colored) and upstream molecules (uncolored) are denoted as follows: ovals, molecules without genomic changes; rectangles with solid line, molecules with copy number deletions, high confidence (p < 0,05 and resistant/parental frequency >30 %) point mutations could not be connected to the network and are thus not displayed. Molecules that are connected with the ER signalling pathway are connected by a dark grey line to the boxed text “Estrogen Receptor Signalling”. (PDF 443 kb) [file 12885_2016_2452_MOESM11_ESM.pdf]

**A** ZR-75-1 Tam1 vs ZR-75-1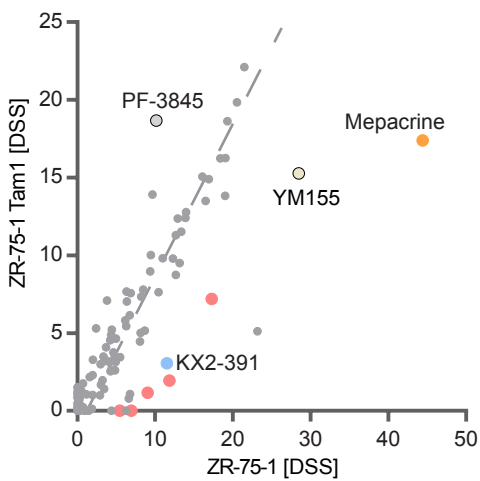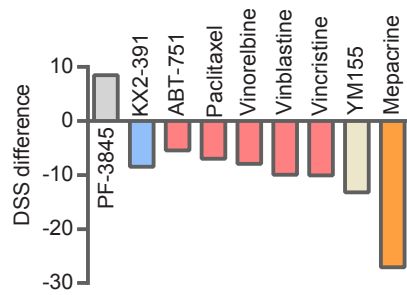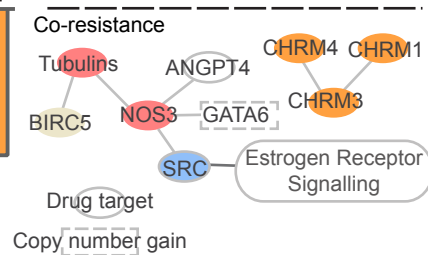**B**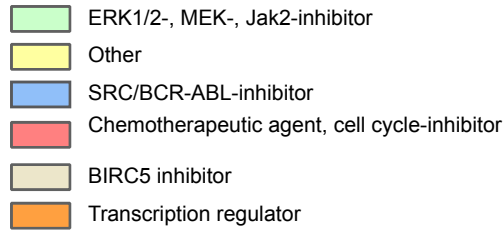**C**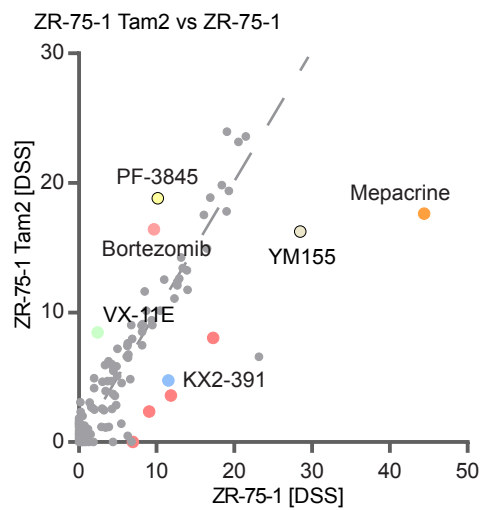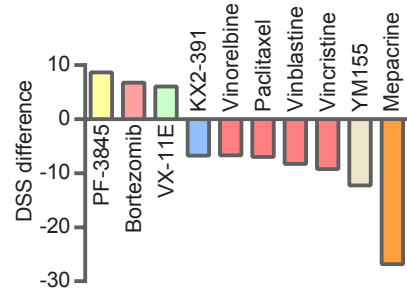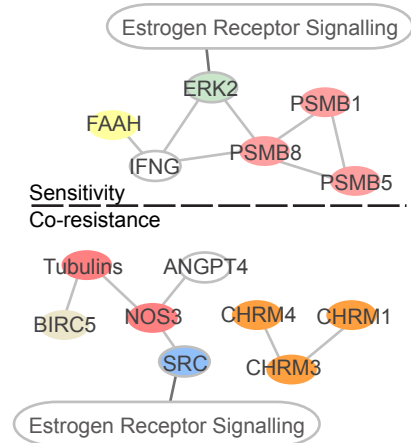

Supplement: Additional file 12: — Drug testing and molecular profiling reveal sensitivity and co-resistance networks in ZR-75-1 Tam1 and Tam2. (left) DSS differences of tamoxifen-resistant (A) ZR-75-1 Tam1 and (B) ZR-75-1 Tam2 vs parental cells reveal emerging sensitivities (above the dotted line) and co-resistances (below the dotted line) upon acquiring tamoxifen resistance. (middle) Drugs with DSS difference >5. Positive values indicate sensitivity and negative co-resistance. (right) Matching of the drugs that the cells show acquired sensitivity or co-resistance towards with their specific target genes reveals molecular networks behind sensitivity and co-resistance in resistant cells. Drugs without target genes in the networks are not displayed. Drug targets (colored) and upstream molecules (uncolored) are denoted as follows: ovals, molecules without genomic changes; rectangles with dashed line, molecules with copy number gain, high confidence (p < 0,05 and resistant/parental frequency >30 %) point mutations could not be connected to the network and are thus not displayed. (B) Color legend of the drug target class. For visualization purposes, the drugs were colored according to their target class as indicated, and the coloring matched with their target genes. Molecules that are connected with the ER signalling pathway are connected by a dark grey line to the boxed text “Estrogen Receptor Signalling”. (PDF 464 kb) [file 12885_2016_2452_MOESM12_ESM.pdf]
